# 

**Published:** 1889-01

**Authors:** 


### CONTRIBUTORS TO VOL. X.

John R. Anderson.
George A. Bodamer, M.D., B.S.
Arthur Erwin Brown.
Richard W. Burke, M.R.C.V.S., A.D.V.
J. S. Butler, V.S.
Halleck S. Campbell.
A. W. Clement, V.S.
Robert Formad, V.D.M.
Matthias L. Forster, M.D.
James H. Frink, G.V.I.
William S. Gottheil, M.D.
Rush Shippen Huidekoper, M.D. Vet.
J. P. Klench, V.S.
Benjamin Lee, AM., M.D., PhD.
Daniel D. Lee, M.D.V.
T. Wesley Mills, M.A., M.D.
Cornelius C. O'Leary, M.D., LL.D.
Jeremiah O'Leary, M.D.
S. C. Orr, V.S.
Leonard Pearson, B. S.
Austin Peters, M.R.C.V.S.
W. H. Ridge, V.D.M.
Walter K. Sibley, M.D.
Richard W. Shufeldt, M.D., C.M.Z.S.
Theobald Smith, M.D.
Edward C. Spitzka, M.D.
J. Bland Sutton, F.R.C.S.
James Vincent, D.V.M.
Kenelm Winslow, B.A.S., M.D.V.

### INDEX.

Abscess of liver . . . . . . . . . . . . . . . . . . . . . . . . . 88
Actinomycosis, the pathology of . . . . . . . . . . . . . . . . . 105, 195
Action of various oils on healthy animals . . . . . . . . . . . . . 180
Address to U. S. veterinary medical association . . . . . . . . . . 332
American Veterinary College . . . . . . . . . . . . . . . . . . . 77, 188
Amputation of uterus in mare . . . . . . . . . . . . . . . . . . . 87
Anderson, J. R., bursatte . . . . . . . . . . . . . . . . . . . . . 73
Animals, tuberculosis in . . . . . . . . . . . . . . . . . . . . . 266
Appropriations for free scholarships . . . . . . . . . . . . . . . 266
Ardeinæ, osteological studies on the subfamily of . . . . . . . . 218, 287

Bears, rickets in . . . . . . . . . . . . . . . . . . . . . . . . . 1
Birds, rickets in . . . . . . . . . . . . . . . . . . . . . . . . . 1
Bodamer, G. A., the pathology of actinomycosis with record of cases and experiments . . . . . . . . . . . . . . . . . . . . . 105, 195
Books and pamphlets received . . . . . . . . . . . . . 102, 191, 283, 407
Brain of parrakeet . . . . . . . . . . . . . . . . . . . . . . . . 164
Bressa prize . . . . . . . . . . . . . . . . . . . . . . . . . . . 165
Burke. R. W., vena azygos in cloven footed animals . . . . . . . . 210
Bursattee . . . . . . . . . . . . . . . . . . . . . . . . . . . . . 73

Camels, an epidemic diarrhœa in . . . . . . . . . . . . . . . . . 172
Campbell, H. S., contribution to the history of congenital intestinal malformation . . . . . . . . . . . . . . . . . . . . . . . . . 169
Case department . . . . . . . . . . . . . . . . . . 87, 167, 269, 374
Cataract in a cat's eye . . . . . . . . . . . . . . . . . . . . . . 167
Circus hudsonius, osteology of . . . . . . . . . . . . . . . . . . 126
Clement, A. W., report on contagious and infectious diseases . . . . 351
Clinical and pathological notes from a breeding station . . . . . . 243
Comparative pathology, society for the study of . . . . . . . . . . 281
Congenital intestinal malformation . . . . . . . . . . . . . . . . 169
Cutaneous horns . . . . . . . . . . . . . . . . . . . . . . . . . . 273
Cystic tumor containing teeth . . . . . . . . . . . . . . . . . . . 270

Diarrhœa in foals . . . . . . . . . . . . . . . . . . . . . . . . . 375
Difficult parturition . . . . . . . . . . . . . . . . . . . . . . . 374
Dog, tuberculosis in a . . . . . . . . . . . . . . . . . . . . . . 169
Domesticated animals, muscular coats of the œsophagus of . . . . . . 59

Editorial department . . . . . . . . . . . . . . . . 81, 162, 263, 382
Epithelioma in the horse . . . . . . . . . . . . . . . . . . . . . 274
Eye, displacement of in the horse . . . . . . . . . . . . . . . . . 91

Foals, diarrhœa in . . . . . . . . . . 375
Foster, M. L., extraction of a cataract luxated into the anterior chamber
of a cat's eye . . . . . . . . . . 167
Fracture of os suffraginis . . . . . . . . . . 87
Frink, J. H., abscess of liver . . . . . . . . . . 88
amputation of uterus in a mare . . . . . . . . . . 87
diarrhœa in foals . . . . . . . . . . 375
difficult parturition . . . . . . . . . . 374
fracture of os suffraginis . . . . . . . . . . 87
post-partum—paralysis . . . . . . . . . . 376
General lymphangitis . . . . . . . . . . 360

Gottheil, W. S., pentastoma moniliforma, a rare parasite with remarks
on the pentastomidæ . . . . . . . . . . 42

Heifer, molluscoid fibromata in a . . . . . . . . . . 171
Horn on goat's ear . . . . . . . . . . 165
Horse, epithelioma in the . . . . . . . . . . 274
lecture on emergencies and their treatment . . . . . . . . . . 247
military supply . . . . . . . . . . 165
transmissibility of colors in . . . . . . . . . . 162
upward displacement of the eye . . . . . . . . . . 91
Huidekoper, R. S., address to the U. S. veterinary medical association 332
lecture on the emergencies of the horse and their
treatment . . . . . . . . . . 247
rabies . . . . . . . . . . 46

International congress of veterinary medicine . . . . . . . . . . 267
Iowa state veterinary medical association . . . . . . . . . . 393

Klench, J, P., general lymphangitis . . . . . . . . . . 360

Laryngotomy for the cure of roaring . . . . . . . . . . 160
Lecture on emergencies of the horse and their treatment . . . . . . . . . . 247
Lee, B., upward displacement of the eye in the horse . . . . . . . . . . 91
Lee, D. D., laryngotomy for the cure of roaring . . . . . . . . . . 160
Luxation of perforatus tendon from os calcis . . . . . . . . . . 269
Lymphangitis, general . . . . . . . . . . 360

Mare, amputation of uterus in . . . . . . . . . . 87
prolonged gestation in a . . . . . . . . . . 270
Military horse supply . . . . . . . . . . 165
Mills, T. Wesley, clinical and pathological notes from a breeding
station . . . . . . . . . . 243
Molluscoid fibromata in a heifer . . . . . . . . . . 171
Monkeys, rickets in . . . . . . . . . . 1
Montreal veterinary college . . . . . . . . . . 275
Mouse, some observations on coccidia in the renal epithelium of the . 211
Muscular coats of the œsophagus of the domesticated animals . . . . 59

New Jersey state veterinary society . . . . . . . . . . 96, 188, 275, 395

### BOOKS AND PAMPHLETS RECEIVED.

Why Electrolytic Treatment of Stricture does not Succeed in all Hands. By G. C. Meier, M. D. Reprint from Journal of Surgery and Antiseptics. October, 1888.

The Failure of Dr. J. B. Thomas' Treatment of Urethal Stricture by Electrolysis. By Robert Newman, M. D. Reprint from Journal American Medical Associatian. September, 1888.

The Preferable Climate for Phthisis. By Charles Denison, A. M., M, D. Reprint from Transaction's Ninth International Congress.

Report on the Proceedings of the United States Expedition to Lady Franklin Bay, Grinnell Land. By Adolphus W. Greeley, U. S. A. Vol. I. Washington. 1888.

Report upon Natural History Collections made in Alaska. By Edward W. Nelson. Prepared under the direction of the Chief Signal Officer. Washington. 1887.

Report of the Ornithologist. Dr. C. Hart Merriam of the Agricultural Department. Washington. 1888.

Report on Bird Migration in the Mississippi Valley. By W. W. Cooke. Bulletin No. 2, U. S. Department Agriculture.

Guide to a Collection Illustrating the Families of Mammals, exhibited in the Ohio Valley Centennial Exposition in 1888. By Frederick W. True, Curator, U. S. National Musuem.

Contributions to the Comparative Osteology of Arctic and sub-Arctic Water Birds, Part I. By R. W. Shufeldt, M. D. Reprint from Journal of Anatomy and Physiology, Vol. XXIII.

The Osteology of Habia Melanocephala. By R. W. Shufeldt, M. D. Reprint from the Auk. October, 1888.

Constitution, By-Laws, Officers and Members for 1888–9 of the American Pediatric Society. Philadelphia: J. B. Lippincott & Co. 1888.

Annual Report of the President to the Corporation of Brown University. Providence. 1888.

*Albany.*—Medical Annals.

*Amiens.*—Bulletin Mensuel Societe Linneene de Nord de la France.

*Augsburg.*—Wochenschrift fur Theirheilkunde und Viehzucht.

*Baltimore.*—Johns Hopkins University Circulars.

*Brisbane.*—Proceedings Royal Society of Queensland. Vol. V, Pt. 1–2.

*Brussels.*—Bulletin Musee Royal d' Historie Naturelle de Belgique. Annales de Medicine Veterinaire.

*Calcutta.*—Journal Asiatic Society of Bengal. Vol. LVII, Pt. II, Nos. 1–3.

*Cap Rouge.*—Le Naturaliste Canadien.

*Chicago.*—Weekly Drovers' Journal, National Live Stock Journal, Herds and Flocks.

*Cincinnati.*—Journal Society Natural History.

*Copenhagen.*—Tidsskrift fur Veterinærer.

*Detroit.*—The Microscope.

*Edinburgh.*—Journal of Comparative Pathology and Therapeutics.

*Frankfort.*—Der Zoologische Garten.

*Ghent.*—Annales et Bulletin de la Societe de Medicine.

*Glasgow.*—Proceedings and Transactions of the Natural History Society. Vol. II. Pt. 1.

*Jena.*—Jenatsche Zeitschrift fur Natur-wissenschaft von der Medizinisch Natur-wissenschaftlichen Gesellschaft. Bd. XXII, Heft 3-4.

*Karlsruhe.*—Thieraztliche Mittheilungen. Amtliche Bekamlmachungen uber das Veterinarwesen in Grossherzogthum.

*Knoxville.*—Agricultural Science.

*London.*—The Veterinary Journal. The Veterinarian.

*Lyon.*—Journal de Medecine Veterinaire et de Zootechnie.

*Madrid.*—Gaceta Medico-Veterinaria.

*Milan.*—La Clinica Veterinaria.

*Montreal.*—Canadien Record of Science.

*Munich.*—Sitzungsberichte der mathematish-physikalischen classe der K. B. Akademie der wissenschaften. Heft 1-2. 1888.

*New York.*—Medical Analectic, New York Medical Journal, Scientific American, Forrest and Stream, American Veterinary Review.

*Ottawa.*—Ottawa Naturalist.

*Palermo.*—Il Naturalesta Siciliano.

*Paris*—Le Progres Medical, Recueil de Medecine Veterinaire, Bulletin de la Societe Zoologique de France.

*Philadelphia.*—American Naturalist, Medical and Surgical Reporter, Therapeutic Gazette, Reins and Whip.

*Red Wing.*—Public Health in Minnesota.

*San Jose.*—Anales del Museo Nacional Tomo I.

*St. Louis.*—Weekly Medical Review.

*St. Petersburg.*—Archives of Veterinary Science.

*Stockholm.*—Tidskrift for Veterinar-Medicin.

*Stuttgart.*—Repertorium der Thielkunde,—Hippologische Revue.

*Sydney.*—Journal and Proceedings Royal Society New South Wales, Vol. xxii, Pt. I. Proceedings of the Linnean Society of New South Wales, Vol. III., Pt. II.

*Toronto.*—Proceedings Canadien Institute, Vol. 6. Fas. I.

*Toulouse.*—Revue Veterinarie.

*Udine.*—Giornale de Veterinaria Militare.

*Valencia.*—La Cronica Medica.

*Vienna.*—Mittheilungen des Ornithologischen Vereins,— Oesterreichische Monatschrift fur Theirheilkunde,—Ornis.

*Wilmington.*—Delaware Farm and Home.

*Zurich.*—Schweizer-Archiv fur Thierheilkunde.

